# Supplementary material for: Unveiling the urban sports landscape: Profiling participants, motives, and policy implications
Source: PLoS One. 2024 Jul 5;19(7):e0306305. doi: 10.1371/journal.pone.0306305 (PMC11226041; doi:10.1371/journal.pone.0306305)
Supplement: Attachment — Submitted filename: PONE-D-24-02881_Revisado.pdf [file pone.0306305.s002.pdf]

PLOS ONE

Unveiling the urban sports landscape: profiling participants, motives, and policy implications

--Manuscript Draft--

**Manuscript Number:**	PONE-D-24-02881
**Article Type:**	Research Article
**Full Title:**	Unveiling the urban sports landscape: profiling participants, motives, and policy implications
**Short Title:**	Urban sports participation
**Corresponding Author:**	Jef Huyghe, M.D.<br>KU Leuven: Katholieke Universiteit Leuven<br>Leuven, BELGIUM
**Keywords:**	urban sports, action sports, lifestyle sports, physical activity, sports participation, sportification, sportisation, sports policy
**Abstract:**	BackgroundThe lack of knowledge regarding urban sports poses pressing challenges for governments and sports organisations to deal with in light of its increasingpopularity. To develop targeted policy strategies, more insight is needed into the features of urban sports. Therefore, this research aims to establish a profile of urban sports participants in terms of characteristics, behaviours in sports participation, and motives for sports participation.MethodsData was gathered during a large-scale research project on sports participation in the Belgian city of Bruges. A total of 3,951 residents between 6 and 75 years old participated in an online survey. Descriptive statistics and binomial logistic regression analyses were used to examine the differences between urban and traditional sports participants.ResultsBased on a binomial regression model, the sports level and location significantly predict urban sports participation. The physical motives for sports participation are significantly less important for urban sports participants compared to the group of traditional sports participants. Urban sports participants value the fun and relaxation motives for sports participation significantly more than traditional sports participants. ConclusionsThis paper aimed to establish a comprehensive profile of urban sports participants and juxtaposed it to that of traditional sports participants. While existing literature often portrays urban sports participants as a relatively homogeneous group, our findings reveal a surprising heterogeneity. Consequently, given its increasing popularity, urban sports present a valuable avenue for governments and sports organisations to engage with a diverse range of sports participants.
**Order of Authors:**	Jef Huyghe, M.D.
	Nathan D'hoore, M.D.
	Erik Thibaut, D.D.
	Jeroen Scheerder, Professor
**Additional Information:**	
**Question**	**Response**
**Financial Disclosure**<br><br>Enter a financial disclosure statement that describes the sources of funding for the work included in this submission. Review the submission guidelines for detailed requirements. View published research articles from *PLOS ONE* for specific examples.	Yes

Powered by Editorial Manager® and ProduXion Manager® from Aries Systems Corporation

This statement is required for submission and **will appear in the published article** if the submission is accepted. Please make sure it is accurate.<br><br>**Funded studies**<br>Enter a statement with the following details:<br>• Initials of the authors who received each award<br>• Grant numbers awarded to each author<br>• The full name of each funder<br>• URL of each funder website<br>• Did the sponsors or funders play any role in the study design, data collection and analysis, decision to publish, or preparation of the manuscript?<br><br>Did you receive funding for this work?	
Please add funding details.<br>as follow-up to "**Financial Disclosure**<br><br>Enter a financial disclosure statement that describes the sources of funding for the work included in this submission. Review the submission guidelines for detailed requirements. View published research articles from *PLOS ONE* for specific examples.<br><br>This statement is required for submission and **will appear in the published article** if the submission is accepted. Please make sure it is accurate.<br><br>**Funded studies**<br>Enter a statement with the following details:<br>• Initials of the authors who received each award<br>• Grant numbers awarded to each author<br>• The full name of each funder<br>• URL of each funder website<br>• Did the sponsors or funders play any role in the study design, data collection and analysis, decision to publish, or preparation of the manuscript?	The funders had no role in study design, data collection and analysis, decision to publish, or preparation of the manuscript.

Powered by Editorial Manager® and ProduXion Manager® from Aries Systems Corporation

Did you receive funding for this work?"	
Please select the country of your main research funder (please select carefully as in some cases this is used in fee calculation).<br>as follow-up to "**Financial Disclosure**<br><br>Enter a financial disclosure statement that describes the sources of funding for the work included in this submission. Review the submission guidelines for detailed requirements. View published research articles from *PLOS ONE* for specific examples.<br><br>This statement is required for submission and **will appear in the published article** if the submission is accepted. Please make sure it is accurate.<br><br>**Funded studies**<br>Enter a statement with the following details:<br>• Initials of the authors who received each award<br>• Grant numbers awarded to each author<br>• The full name of each funder<br>• URL of each funder website<br>• Did the sponsors or funders play any role in the study design, data collection and analysis, decision to publish, or preparation of the manuscript?<br><br>Did you receive funding for this work?"	BELGIUM - BE
**Competing Interests**<br><br>Use the instructions below to enter a competing interest statement for this submission. On behalf of all authors, disclose any competing interests that could be perceived to bias this work—acknowledging all financial support and any other relevant financial or non-financial competing interests.<br><br>This statement is required for submission and **will appear in the published article** if	On behalf of all authors I disclose any competing interests that could be perceived to bias this work.

Powered by Editorial Manager® and ProduXion Manager® from Aries Systems Corporation

the submission is accepted. Please make sure it is accurate and that any funding sources listed in your Funding Information later in the submission form are also declared in your Financial Disclosure statement.<br><br>View published research articles from *PLOS ONE* for specific examples.<br><br>**NO authors have competing interests**<br><br>Enter: *The authors have declared that no competing interests exist.*<br><br>**Authors with competing interests**<br><br>Enter competing interest details beginning with this statement:<br><br>*I have read the journal's policy and the authors of this manuscript have the following competing interests: [insert competing interests here]*<br><br>* typeset	
**Ethics Statement**<br><br>Enter an ethics statement for this submission. This statement is required if the study involved:<br><br>• Human participants<br>• Human specimens or tissue<br>• Vertebrate animals or cephalopods<br>• Vertebrate embryos or tissues<br>• Field research<br><br>Write "N/A" if the submission does not require an ethics statement.<br><br>General guidance is provided below. Consult the submission guidelines for detailed instructions. **Make sure that all information entered here is included in the Methods section of the manuscript.**	Ethical guidelines were followed and ethical approval for the data collection was given on 25th of April 2022 by the Sociaal-Maatschappelijke Ethische Commissie (SMEC) of the KU Leuven (G-2022-4993-R2(AMD)). An informed consent was signed by all participants.

Powered by Editorial Manager® and ProduXion Manager® from Aries Systems Corporation

**Format for specific study types**<br><br>**Human Subject Research (involving human participants and/or tissue)**<br>• Give the name of the institutional review board or ethics committee that approved the study<br>• Include the approval number and/or a statement indicating approval of this research<br>• Indicate the form of consent obtained (written/oral) or the reason that consent was not obtained (e.g. the data were analyzed anonymously)<br><br>**Animal Research (involving vertebrate animals, embryos or tissues)**<br>• Provide the name of the Institutional Animal Care and Use Committee (IACUC) or other relevant ethics board that reviewed the study protocol, and indicate whether they approved this research or granted a formal waiver of ethical approval<br>• Include an approval number if one was obtained<br>• If the study involved *non-human primates*, add *additional details* about animal welfare and steps taken to ameliorate suffering<br>• If anesthesia, euthanasia, or any kind of animal sacrifice is part of the study, include briefly which substances and/or methods were applied<br><br>**Field Research**<br><br>Include the following details if this study involves the collection of plant, animal, or other materials from a natural setting:<br>• Field permit number<br>• Name of the institution or relevant body that granted permission	
**Data Availability**<br><br>Authors are required to make all data underlying the findings described fully available, without restriction, and from the time of publication. PLOS allows rare exceptions to address legal and ethical concerns. See the PLOS Data Policy and FAQ for detailed information.	Yes - all data are fully available without restriction

A Data Availability Statement describing where the data can be found is required at submission. Your answers to this question constitute the Data Availability Statement and **will be published in the article**, if accepted.<br><br>**Important:** Stating 'data available on request from the author' is not sufficient. If your data are only available upon request, select 'No' for the first question and explain your exceptional situation in the text box.<br><br>Do the authors confirm that all data underlying the findings described in their manuscript are fully available without restriction?	
**Describe where the data may be found in full sentences. If you are copying our sample text, replace any instances of XXX with the appropriate details.**<br><br>• If the data are **held or will be held in a public repository**, include URLs, accession numbers or DOIs. If this information will only be available after acceptance, indicate this by ticking the box below. For example: *All XXX files are available from the XXX database (accession number(s) XXX, XXX.).*<br>• If the data are all contained **within the manuscript and/or Supporting Information files**, enter the following: *All relevant data are within the manuscript and its Supporting Information files.*<br>• If neither of these applies but you are able to provide **details of access elsewhere**, with or without limitations, please do so. For example:<br><br>*Data cannot be shared publicly because of [XXX]. Data are available from the XXX Institutional Data Access / Ethics Committee (contact via XXX) for researchers who meet the criteria for access to confidential data.*<br><br>*The data underlying the results presented in the study are available from (include the name of the third party*	All relevant data are within the manuscript and its Supporting Information files.

and contact information or URL).<br>• This text is appropriate if the data are owned by a third party and authors do not have permission to share the data.<br><br>* typeset	
Additional data availability information:	

Manuscript

Click here to access/download;Manuscript;Paper urban sports.docx

Unveiling the urban sports landscape: profiling participants, motives, and policy implications

Urban sports participation

Jef Huyghe[1], Nathan D'Hoore[1], Erik Thibaut[1], Jeroen Scheerder[1]

[1]Departement of Movement Sciences, KU Leuven, Leuven, Belgium

Abstract

Background

The lack of knowledge regarding urban sports poses pressing challenges for governments and sports organisations to deal with in light of its increasing popularity. To develop targeted policy strategies, more insight is needed into the features of urban sports. Therefore, this research aims to establish a profile of urban sports participants in terms of characteristics, behaviours in sports participation, and motives for sports participation.

Methods

Data was gathered during a large-scale research project on sports participation in the Belgian city of Bruges. A total of 3,951 residents between 6 and 75 years old participated in an online survey. Descriptive statistics and binomial logistic regression analyses were used to examine the differences between urban and traditional sports participants.

Results

Based on a binomial regression model, the sports level and location significantly predict urban sports participation. The physical motives for sports participation are significantly less important for urban sports participants compared to the group of traditional sports participants. Urban sports participants value the fun and relaxation motives for sports participation significantly more than traditional sports participants.

Conclusions

This paper aimed to establish a comprehensive profile of urban sports participants and juxtaposed it to that of traditional sports participants. While existing literature often portrays urban sports participants as a relatively homogeneous group, our findings reveal a surprising heterogeneity.

Consequently, given its increasing popularity, urban sports present a valuable avenue for governments and sports organisations to engage with a diverse range of sports participants.

Introduction

Since sport is regarded as an important driver of social and economic well-being, it is high on the agenda of local governments and political bodies [1,2,3]. Specifically, governments – mainly in Western European countries – strive to promote and facilitate sports participation among the non-sporting population and to prevent the interest of sports participants from being lost [4]. By increasing sports participation, governments target to obtain beneficial effects on the population such as social inclusion, physical health and mental health, among other things [5,6]. Although sports participation in Western Europe has increased constantly over the past decades, this trend appears to have stagnated in recent years [7,8,9,10]. According to sports participation surveys in Flanders – the region of interest in this research – in 2009, 2014 and 2020, sports participation figures stagnated; respectively 62.8%, 62.7%, and 61.2% of the Flemish population between 15 and 85 years old were active in sports at least once a year [9]. At the same time, the observed sports participation is subject to diversification, changing socio-cultural contexts, and social transformation [11]. One of the most notable trends in sports is the increasing popularity of less organised and informal sports activities. Specifically in Flanders, these informal sports activities are gaining interest and are now even more popular than club-organised sports which used to be the dominant sport mode for decades [2,12,13,14,15,16]. Moreover, sports behaviour appears to be highly sensitive to social trends and societal changes. For example, the recent Covid-19 pandemic had a global influence on sports participation and physical activity since sports infrastructure was closed, contact sports were prohibited and lockdowns forced people to stay inside. During this period, the literature indicates a decrease in sports participation but also a major shift in the organisational setting of sports [3,17,18]. In essence, due to the Covid-19 pandemic, people were obliged to practise sports in an informal and a non-organised setting and were therefore forced to use their infrastructure at home or public outdoor urban infrastructure. The Covid-19 pandemic accelerated an existing trend towards, what in the literature has been described as, sport-light, informal, or less-organised sports [16,18].

Alongside and within this trend towards more informal sports settings, there is the emerging popularity of so-called action sports and lifestyle sports, such as skateboarding, windsurfing, snowboarding, BMX, etc. Many action sports started to gain interest in the 1960s and 1970s. Action sports originated by participants embracing fun, freedom, and fitness and rejecting the traditional and organised aspects of sports [19,20, 21, 22, 23]. According to Wheaton (2004), participants often regard these action sports activities as a lifestyle rather than as sports [24]. Therefore, lifestyle sports can be considered action sports that are experienced by participants as a lifestyle. Lifestyle sports originated with participants resisting and being co-opted by commercialism and creating their own sporting identities [24]. Ultimately, action and lifestyle sports have become more popular and visible over the past five decades as they are experiencing enormous growth in commercialisation with media exposure and sponsorship, and are increasingly part of marketing strategies [22,25,26]. A prime example of a well-known brand that uses action sports in its promotion is Red Bull. Ranging from major sports events with spectacular tricks in snowboarding, skateboarding, and cliff diving to fixed sponsorship contracts with star athletes in Formula 1, professional cycling, athletics, etc. The Red Bull brand is inextricably associated with action sports [25,26,27]. In addition, major sports events and sports mega-events, as classified by Müller (2015), are arising from these action sports blurring the boundaries between entertainment festivals and sporting events [22,28,29]. This emerging popularity of action and lifestyle sports is also recognised by international sports organisations and institutions. For instance, the International Olympic Committee (IOC) introduced five new action sports, i.e. surfing, skateboarding, 3x3 basketball, sports climbing and BMX freestyle, at the Olympic Games in Tokyo 2020 and has even more action sports scheduled at the Olympics in 2024 and 2028 [30,31,32].

Consistent with the increasing popularity towards action sports and lifestyle sports is the increasing popularity of activities that can be categorised as ‘urban sports’. Specifically, urban sports are defined as free and non-organised sports performed in public spaces, often characterised by a less competitive

and more social environment compared to traditional sports [33,34]. Typical urban sports are skateboarding, BMX, step, parkour/freerunning, 3x3 basket, calisthenics, etc. [35]. However, any non-organised sports activity performed in an urban public space can be regarded as an urban sports activity. For example, football or basketball on a public square or skateboarding in a public park are also considered urban sports activities [33,34]. Ultimately, urban sports fall under the broad scope of non-organised or informal sports. In addition, most urban sports can also be considered action or lifestyle sports. However, since general informal sports activities, action sports, or lifestyle sports do not necessarily have to be performed in the public spaces of cities, the category of urban sports activities is relevant for city governments and policymakers. Due to urban sports' increasing popularity, local governments, especially in bigger cities, are increasingly considering urban sports as an important part of their sports policy strategies [30,35,36,37,38,39,40,41]. For example, in all five capital cities of the provinces in Flanders: Antwerp, Bruges, Hasselt, Gent and Leuven, the policy plan contains a topic on the creation of urban sports space or infrastructure [36,37,39,40,41].

Yet, despite this policy instrumentalization of urban sports, many questions remain unanswered. The lack of knowledge regarding urban sports poses pressing challenges for local governments to deal with in light of its increasing popularity. More specifically, in order to develop targeted policies to promote and facilitate urban sports, more insight is needed into the profile, preferences and main drivers of those who participate in urban sports. Therefore, this research aims to investigate (i) the characteristics of the participants of the urban sports compared to those who only participate in traditional sports, (ii) the general behaviour in sports participation of the urban sports participant compared to the traditional sports participant, and (iii) the motives to participate in urban sports compared to the main motives of traditional sports participants. To examine the difference between the urban sports participant and the traditional sports participant, we draw on data gathered during a larger sports participation study in the Belgian city of Bruges [42]. Bruges is the capital city of the

Belgian province of West Flanders and is one of the thirteen centre cities in Flanders [43]. In the next paragraph, an overview of the existing literature on urban sports will be presented.

Urban sports

As aforementioned, specific literature concerning urban sports is rather scarce. Moreover, an unequivocal definition of urban sports does not exist. Despite the absence of a uniform definition, the most frequently cited characteristics of urban sports are the non-organised setting of the urban sporting activities and their practice in the public space of the city [30,33,34,35,44,45]. The public space used for urban sports activities ranges from public football fields and basketball courts to non-sporting urban infrastructure such as squares, bridges, abandoned buildings, etc. A third, less demarcated characteristic of urban sports is the so-called 'urban culture' [34,44]. Urban culture is described by Van der Meijde and colleagues (2022) as a term for the close-knit community to which participants in urban sports belong, but also for the 'show' element that is typically an important feature of urban sports [34]. According to Van der Meijde and colleagues, this creative part and the urge to push boundaries are the most important distinguishing characteristics of urban sports compared to more traditional sports activities. In addition, the sense of community triumphs over the urge to compete in urban sports [34]. Urban sports participants are argued to value the social aspect and sense of community of their sports activities more than the competitive aspect. The challenge of acquiring skills and the kick of performing tricks is their main drive to practise sports and the social environment is supportive rather than competitive [30,33,34].

In sports policy, urban sports are considered to be of high value to cities and their inhabitants. More specifically, urban sports are believed to be a catalyst for healthy lifestyles and cooperation because of their sporty, non-competitive, and community character [35]. Moreover, the instrumentalisation of urban sports for social benefits is considered to be highly suitable by policymakers for many vulnerable groups in society such as individuals living in poverty and people with a migration background

[21,35,44,46]. In particular, urban sports are considered to reach social groups that are generally difficult to attract because of the low cost of participation and equipment, and the accessible context in the vicinity of cheaper and smaller housing [33,35,47]. Therefore, local governments instrumentalise sports to promote integration and well-being, and to increase participation in the welfare state of relatively underrepresented groups [48]. For instance, in the Belgian city centre of Bruges, the government purchased an old bus station which will be renovated and transformed into an urban sports area with space for parkour/freerunning and boulder, among other things [49]. Moreover, the use of urban sports infrastructure for non-sporting purposes is facilitated by the lower cost price than the use of traditional sports infrastructure. Likewise, urban culture creates new communities of young people in cities that can create a hype image of the city and create economic value as the city becomes more attractive [21,34,35,44,46]. Additionally, the emerging popularity of urban sports makes it an interesting tool for governments in creating a hype image and economic value for their cities. This popularity of urban sports is also recognised by sports organisations and institutions in Flanders. Major urban sports competitions and tournaments are organised in which there is discourse around attracting new participants and fans. For example, the world championships of 3x3 basketball (FIBA 3x3 World Cup) were organised in Antwerp in 2022 and the world championships of breaking (WDSF World Breaking Championship) will be organised in the Flemish city of Leuven in September 2023 [50,51].

This institutionalisation and formalisation of urban sports is often referred to as 'sportisation' [52,53]. Specifically, 'sportisation' is the term used to describe the process of incorporating 'play-like' activities into the structure of sports, transforming them into more competitive, regularised, and rationalised endeavours [54,55]. Urban sports are going through organisational development, adopting rules and structures [52,53]. For example, Larsen (2022) delineated the evolution of parkour in Denmark from its origins as a self-organised and play-centric pursuit to a structured discipline marked by weekly training sessions, formal associations, and dedicated sports facilities [52,53].

Materials and methods

Data collection

The data used in this paper were collected as part of a larger research project on sports participation within the population of the Belgian city of Bruges. The goal of this study was to acquire insight into the sports and physical activity behaviour of residents in Bruges. For a detailed methodology of the broader research project on sports participation in Bruges, we refer to the work of Scheerder and Huyghe (2023) [42]. The method relevant to this paper is described below. A total of 25,449 inhabitants of Bruges aged 6 to 75 years were sampled out of an anonymised city register and were contacted by letter to participate in an online survey concerning their participation in sports and physical activity. The inhabitants of Bruges were randomly selected, based on seven demographic and social characteristics (gender, age, level of education, employment status, nationality, postal code, and family type) to improve representativeness. Qualtrics XM software was used for the survey. The data collection happened from 6/06/2022 until 24/07/2022. After data collection weight coefficients were calculated and applied to ensure representativeness concerning gender, age, and level of education [42]. Ultimately, we obtained a weighted dataset of 3,951 individuals aged 6 to 75 years representative in the city of Bruges according to gender, age and level of education. All data was submitted as part of the paper. Before participating in the survey, participants signed a written informed consent, minors were obliged to have consent from their parents, who in that case had to sign the informed consent.

Ethical guidelines were followed and ethical approval for the data collection was given on 25th of April 2022 by the Sociaal-Maatschappelijke Ethische Commissie (SMEC) of the KU Leuven (G-2022-4993-R2(AMD)). An informed consent was signed by all participants. An official ethical approval document was uploaded as part of this manuscript.

Variables

The larger survey contained twelve themes of which three were used in this paper: (i) socio-demographic characteristics, (ii) sports participation, and (iii) motives to participate in sports. Data with respect to the latter two themes were only collected among participants who practised sports in the past year.

Independent variables

Table 1 shows all the used independent variables in this research, except for the motives for sports participation. As illustrated, socio-demographic characteristics of gender, age, and level of education were used. In addition, the survey contained detailed questions concerning sports participation. Specifically, the following variables with regard to sports participation were utilised in the survey: (i) practised sports, (ii) frequency of sports participation, (iii) time per sports session, (iv) level of sports participation, (v) organisational setting of sports activities, (vi) location of the sports activities, (vii) sports companionship, and (viii) participants' motives to participate in sports.

Table 1: Independent variables of sports participation criteria

Variables	Categories	Description
**Gender**	Men	/
	Women	
**Age**	6-12 years old	/
	13-18 years old	
	19-30 years old	
	31-45 years old	
	46-65 years old	
	66-75 years old	
**Level of education**	Higher education	Higher professional education or university
	Middle education	Secondary education
	Lower education	No education or primary education
	Still in education	Students
		Survey participants were asked to rank their three most practised sports

**Practised sports**	/	activities. After that, the participants were asked to also list other sports they practised in the past year.
**Frequency of sports participation**	Less than once a week (1-27 times per year)	Frequency of sports participation was represented as the number of times per year the participants practised their three main sports.
	Once a week (27-52 times per year)	
	More than once a week (more than 52 times per year)	
**Time per sports session**	Less than one hour per sports session (1-59 min per sports session)	Time per sports session was represented as the average time (in minutes) per sports session.
	One hour or more per sports session (60 min or more per sports session)	
**Level of sports participation**	In a recreational way only	Level of sports participation was examined by asking the respondents if they practise their sports activities in a 'competitive' way, in a 'recreational' way or in a 'competitive and recreational' way. Resulting in three categories for the level at which the sports are practised. Because only 2.4% of the Bruges sportive population practised sports on a competitive level only, these three categories were recoded into two categories.
	In a competitive and recreational way	
**Organisational setting of the sports activities**	Organised sports activities only	Organisational setting of the sports activities was queried for each sports activity separately. Participants had thirteen answer options and multiple answer options could be chosen. These were recoded into two variables.
	Non- or self-organised sports activities	
**Location of sports activities**	In the public space	Location of sports activities was queried for each sports activity separately. Thirteen answer options were possible and multiple answer options could be chosen. These thirteen options were recoded in two new variables. Respondents were
	Never in the public space	

		allocated as 'in the public space' if they practised their sports in a public park on the street on a public square and/or in the forest/outdoors/nature.
**Sports companionship**	At least sometimes alone	Sports companionship was measured by asking in which companions the participants usually practise their sports activities. There were seven answer options with different categories of companions, multiple answer options could be chosen.

In total, sixteen motives to participate in sports were formulated, which the participants had to score on a Likert scale of 1-7 (ranging from 'not applicable to me at all' to 'very applicable to me'). The sixteen motives are listed in Table 2. To reduce the number of variables, a principal component analysis (PCA) was conducted on the sixteen items with orthogonal rotation (varimax). According to Field's (2009), The Kaiser-Meyer-Olkin (KMO) measure of sampling adequacy (KMO = 0.786) was 'good'. All individual KMO values are higher than 0.690, which is well above the acceptable limit of 0.500 [56]. Given the significant result of Bartlett's test of sphericity ($p<0.001$), there were sufficient relationships between the variables which makes a factor analysis appropriate. An analysis to obtain eigenvalues for each component showed four components with an eigenvalue higher than one. These four components explained in total 58.86% of the variance. Since the selection of four components was supported by the scree plot, four components were retained for the final analysis. Table 2 shows the sixteen motives with their rotated factor loadings per cluster. The clustered motives lead to the following four components: (i) physical motives, (ii) social motives, (iii) satisfaction and recognition motives, and (iv) fun and relaxation motives. Reliability analysis showed good reliability scores for the physical motives (α=0.78), the social motives (α=0.76) and the satisfaction and recognition motives (α=0.68). However, the fun and relaxation motives had relatively low reliability (α=0.47). Four new variables of clustered motives were created by calculating the average score of the corresponding motives. Each motive was allocated to the cluster with their highest rotated factor loading which is shown in bold in Table 2.

Table 2: Summary of exploratory factor analysis results for the sixteen motives to participate in sports (N= 2,642)

Motives to participate in sports	Physical motives	Social motives	Satisfaction and recognition motives	Fun and relaxation motives
My health is improving	**0.812**			
My body becomes more beautiful	**0.755**			
My physical condition is improving	**0.754**			
I lose weight	**0.714**			
I get to know new people		**0.737**		
I am with my friends		**0.717**		
It makes me part of society		**0.707**		
Others stimulated me		**0.620**		
It is an opportunity to network		**0.562**	0.486	
Others look up to me			**0.674**	
I earn money			**0.597**	
I feel the kick			**0.591**	0.539
It is a compensation for my hard work-life			**0.548**	
I can compete			**0.540**	0.452
I have fun				**0.768**
I feel less tension, stress, sadness, or aggression	0.401			**0.509**
**Eigenvalues**	2.644	2.481	2.333	1.961
**% of variance**	16.524	15.506	14.578	12.256
α	0.782	0.760	0.678	0.471

Only factor loadings over 0.40 are shown.
The highest factor loading per motive appears in bold.

Dependent variables: urban sports participants and non-urban sports participants

To measure urban sports participation a new variable was created since urban sports participation was not directly queried in the survey. As described in the introduction, there is no unequivocal definition of urban sports. Therefore, two different approaches to conceptualising urban sports were used in this paper.

First, sports were classified as urban sports based on the specific sport. The following sixteen sports were allocated as urban sports based on (non-exhaustive) lists in literature: 3x3 basket, BMX, breaking, calisthenics, parkour/freerunning, inline/stuntstep, pétanque, skateboard, tricking, bootcamp, boulder, headis, inlineskating, slacklining, tai chi, and urban roundnet (spikeball) [35,57].

Second, a broader definition of urban sports was used. Individuals who practised sports in a non-organised setting and public space were defined as urban sports participants. For example, in this definition, a person playing football in a public park with friends is considered an urban sports participant.

Data analysis

SPSS 28.0.1.1 was used for statistical analysis. First, descriptive statistics were calculated, and Chi-squares were conducted to test for significant differences between sports participants and non-sports participants and between sports participants in general and urban sports participants. Secondly, three binomial regression analyses were conducted. First, to get insight into the differences in general population characteristics between the sporting and the non-sporting population in Bruges. Then, two binomial regression analyses were carried out to examine the differences between urban sports participants and (more) traditional sports participants (non-urban sports participants). This analysis was conducted twice using the two abovementioned definitions of urban sports. These two binomial regression analyses to examine the differences between urban sports participants and non-urban sports participants contained three blocks. The first block consisted of socio-demographic characteristics (gender, age, educational level), the second block contained sports participation

(frequency, time per sports session, level, setting, location, companionship) and the third block contained the four motivations to practise sports (physical motives, social motives, satisfaction and recognition motives, and fun and relaxation motives). In the analysis where urban sports participants were defined based on the setting and the location of their sports participation, the setting and the location of sports were excluded to avoid linearity, since the dependent variable was based on these two criteria.

Results

Descriptive statistics

Total population

The descriptive statistics are listed in Table 3. The weighted dataset contains 3,951 Bruges civilians of which 74.7% participated in sports in the past year. Women represent 50.8% of the total dataset, men represent 49.2%. Almost a third of the respondents (31.2%) have a high educational level and 20.8% of the respondents are still in education.

Sports population

In the sports-active population, men (51.1%) are slightly more strongly represented than women (48.9%). The highly educated represent 33.4% of the sports-active population.

Most of the sports participants (69.1%) practise sports more than once a week, and more than half of the respondents (50.9%) have sports sessions of one hour or more. Most sports participants practise their sports exclusively in a recreational way (70.3%) and in a non-organised setting (73.9%). A little more than a quarter of the sport's participants (25.6%) go to the public space to practise their sports, and 39.4% of the participants indicate to sometimes practise their sports without companionship.

The fun and relaxation motives have the highest mean score with a mean score of 5.52 followed by the physical motives (4.74), the social motives (3.26), and the satisfaction and recognition motives (2.69).

Urban sports population (based on specific sports)

The urban sports participants defined by their specific sports compared to the total sports population are significantly more often men (60.8%) and are generally younger (12.9% aged 6-12 years, 14.0% aged 13-18 years, 24.6% aged 19-30 years). The age group of 46-65 years old is significantly less represented in the urban sports population. Urban sports practitioners are significantly more often students (33.1%). Moreover, urban sports participants have significantly longer sports sessions (63.3% more than one hour per session), are more competitively (41.8% competitive and/or recreational), and practise their sports more in the public space (52.1% in public space) compared to non-urban sports participants. Also, urban sports participants practise their sports significantly more in a non-organised setting (83.4%).

Urban sports population (defined by setting and location of sports participation)

The urban sports participants defined by the setting and location of their sports participation show less significant differences in sports participation criteria. Apart from differences in sports location and sports setting, urban sports participants practise their sports significantly more 'more than once a week' (78.9%) and significantly less 'once a week' (7.9%). Concerning socio-demographic characteristics, the age group of 19-30 years old (23.9%) and the low-educated people (17.2%) are significantly more represented in the urban sports population, and the age group of 46 to 65 years old (24.6%) is less represented in the urban sports population.

Table 3: Descriptive statistics of the variables

	Total population (N=3,951)	Sports population (N=2,950)	Urban sports population (based on specific sports) (N=160)	Urban sports population (based on location and setting) (N=755)
**Gender (%)**				
Men	49.2	51.1	60.8*	53.5
Women	50.8	48.9	39.2*	46.5
**Age (%)**				
6-12y	8.5	10.6	12.9	9.1
13-18y	7.9	9.6	14.0	10.7
19-30y	16.8	19.9	24.6	23.9**
31-45y	21.4	23.1	23.4	22.7
46-65y	33.2	28.2	12.9**	24.6*
66-75y	12.1	8.6	12.3	8.9
**Educational level (%)**				
High	31.2	33.4	28.5	31.2
Medium	28.8	26.7	22.7	25.4
Low	19.3	14.3	15.7	17.2*
Still in education	20.8	25.6	33.1*	26.3
**Sports frequency (%)**				
Less than once a week	-	14.3	11.8	13.1
Once a week	-	13.7	15.3	7.9**
More than once a week	-	69.1	72.3	78.9**
Missing	-	2.9	/	/
**Time per sports session (%)**				
Less than one hour per session	-	46.0	36.7**	48.2
One hour or more per session	-	50.9	63.3**	51.8
Missing	-	3.0	/	/

**Sports level (%)**				
Always recreational	-	70.3	58.2**	69.7
Recreational and/or competitive	-	26.7	41.8**	30.3
Missing	-	2.9	/	/
**Sports setting (%)**				
Organised only	-	23.1	16.6*	0.0**
Non- /self-organised	-	73.9	83.4*	100.0**
Missing	-	2.9	/	/
**Sports location (%)**				
In public space	-	25.6	52.1**	100.0**
Never in public space	-	71.4	47.9**	0.0**
Missing	-	2.9	/	/
**Sports companionship (%)**				
At least sometimes alone	-	39.4	36.3	39.8
Always with companions	-	57.7	63.7	60.2
Missing	-	2.9	/	/
**Motives mean (SD)**				
Physical motives	-	4.74 (1.28)	4.51 (1.24)	4.81 (2.22)
Social motives	-	3.26 (1.29)	3.60 (1.09)	3.41 (3.31)
Satisfaction and recognition motives	-	2.69 (1.18)	2.91 (1.18)	2.85 (1.21)
Fun and relaxation motives	-	5.52 (1.17)	5.75 (1.03)	5.61 (1.10)

*$p<0.05$, **$p<0.01$

Differences between urban sports participants and (more) traditional sports participants (non-urban sports participants)

Urban sports population (based on specific sports)

Table 4 shows the results of the binomial regression analysis in which urban sports participants are compared to sports participants who do not participate in urban sports. In this model, the urban sports participants were defined based on the specific sports they practised in the past year. The first model with general population characteristics does not explain a lot of variance with a low Nagelkerke R^2 = 0.035. Men (exp(β)=1.461) are significantly more likely to participate in urban sports compared to women within the sports-active population. Sports participants aged 46 to 65 years old are significantly less likely to participate in urban sports compared to the youngest age group of 6 to 12 years old. However, the low Nagelkerke R^2 implies that the general population characteristics in this model can only contribute to a minor predictive value for urban sports participation.

In the second model, shown in Table 4, the sports participation criteria are added. This results in a higher Nagelkerke R^2 of 0.106. The age group of 46 to 65 years old is still significantly less likely to participate in urban sports (exp(β)=0.316) than the youngest age group. All other general population characteristics have no significant value in the model.

In terms of sports participation characteristics, urban sports participants have significantly more often sports sessions of one hour or more (exp(β)=1.456) and practise their sports significantly more in a competitive way and/or a recreational way (exp(β)=1.638) instead of a recreational way. Ultimately, the location of sports shows significant differences as well. Urban sports participants, based on their specific sports, practise their sports more often in a public location (exp(β)=3.272) compared to the non-urban sports participants. With a Wald statistic of 44.765, the sports location is by far the best predictor of urban sports.

Adding the motives for sports participation in the third model, the Nagelkerke R^2 increases to 0.119. The age group of 46 to 65 years old is still significantly less likely to participate in urban sports (exp(β)=0.354) compared to the youngest age group. All other general population characteristics

show no significant value. The differences in time per sports session explain no longer a significant amount of variance. The sports level and the sports location have still a significant role in predicting urban sports participation with an exp(β)=1.711 for sporting in a competitive way and a recreational way instead of a recreational way only, and an exp(β)=3.256 for practising sports in a public location. The physical motives are significantly less important for urban sports participants than for non-urban sports participants (exp(β)=0.844). Urban sports participants value the fun and relaxation motives for sports participation significantly more with an exp(β) of 1.246. According to the Wald statistic, the sports location is still the highest contributor to the model (44.190). The fun and relaxation motives are the second-best predictors with a Wald of 6.082 followed by the sports level (6.038), the physical motive (5.096) and the age group of 46-65 years old (4.109).

Table 4: Binomial logistic regression analysis of urban sports participants and (more traditional) sports participants (N=2,635)

	Model 1: (general population characteristics)			Model 2: (sports participation criteria)			Model 3: (motives for sports participation		
	β (S.E.)	Wald	Exp(β)	β (S.E.)	Wald	Exp(β)	β (S.E.)	Wald	Exp(β)
**Gender (women=ref)**									
Men	0.379* (0.168)	5.078	1.461*	0.240 (0.177)	1.845	1.272	0.225 (0.178)	1.596	1.253
**Age (6-12y=ref)**									
13-18y	0.114 (0.320)	0.127	1.121	-0.095 (0.335)	0.080	0.909	0.028 (0.343)	0.007	1.028
19-30y	0.016 (0.378)	0.002	1.016	-0.182 (0.398)	0.210	0.833	0.071 (0.416)	0.029	1.073
31-45y	-0.345 (0.452)	0.581	0.708	-0.402 (0.475)	0.716	0.669	-0.197 (0.490)	0.162	0.821
46-65y	-1.123*(0.475)	5.577	0.325*	-1.151*(0.498)	5.344	0.316*	-1.039*(0.513)	4.109	0.354*
66-75y	0.078 (0.490)	0.025	1.081	0.035 (0.514)	0.005	1.036	0.107 (0.528)	0.041	1.113
**Educational level (high education=ref)**									
Medium	-0.166 (0.233)	0.505	0.847	-0.142 (0.237)	0.358	0.868	-0.157 (0.240)	0.427	0.855
Low	0.228 (0.279)	0.664	1.256	0.058 (0.285)	0.041	1.059	0.072 (0.291)	0.062	1.075
Still in education	-0.031 (0.371)	0.007	0.970	-0.092 (0.381)	0.059	0.912	-0.149 (0.385)	0.149	0.862
**Frequency (less than once a week=ref)**		-							
Once a week	-	-	-	0.485 (0.321)	0.421	1.625	0.440 (0.324)	0.423	1.553
More than once a week	-	-	-	-0.174 (0.268)	2.286	0.840	-0.177 (0.272)	1.851	0.838
**Time per sports session (less than one hour per session=ref)**		-							
One hour or more per session	-	-	-	0.375*(0.182)	4.232	1.456*	0.343 (0.184)	3.466	1.409
**Level (only in a recreational way =ref)**		-							
In a competitive and a recreational way	-	-	-	0.493*(0.196)	6.301	1.638*	0.537*(0.219)	6.038	1.711*

**Organisational setting (only organised sports settings=ref)**		-							
At least sometimes non- or self-organised	-	-	-	0.338 (0.246)	1.887	1.402	0.341 (0.246)	1.922	1.406
**Location (never in public space)**		-							
In public space	-	-	-	1.185**(0.177)	44.765	3.272**	1.181**(0.178)	44.190	3.256**
**Companionship (at least sometimes alone)**		-							
Only in companionship	-	-	-	0.134 (0.185)	0.526	1.144	0.034 (0.189)	0.033	1.035
**Motives for sports participation**									
Physical motives<br>Social motives<br>Satisfaction and recognition motives<br>Fun and relaxation motives	-<br>-<br>-<br>-	-<br>-<br>-<br>-	-<br>-<br>-<br>-	-<br>-<br>-<br>-	-<br>-<br>-<br>-	-<br>-<br>-<br>-	-0.170*(0.075)<br>0.146 (0.079)<br>-0.164 (0.100)<br>0.220*(0.089)	5.096<br>3.394<br>2.693<br>6.082	0.844*<br>1.157<br>0.849<br>1.246*
**Model summary**									
Nagelkerke R^2<br>Chi-square<br>-2 Log likelihood<br>Cox & Snell R Square	0.035<br>34.091**<br>1171.096<br>0.013			0.106<br>104.268**<br>1100.919<br>0.039			0.119<br>118.071**<br>1087.116<br>0.044		

Dependent variables: sports participants practising urban sports (N=160); sports participants practising other (non-urban) sports (N=2,475).
**p<0.05, **p<0.01*

Urban sports population (defined by setting and location of sports participation)

The results of the regression analysis of the larger definition of urban sports, defined by the sports location and the sports setting (N=755) are not included in this paper. The Nagelkerke R^2 of 0.050 after adding general population characteristics, sports participation, and motives indicates a rather low predictive value of the model. Also, few significant factors for predicting urban sports participation were established.

Discussion

The results presented provide critical insights into the characteristics and motives of urban sports participants in Bruges, Belgium. We observed significant disparities between urban and traditional sports participants across various demographics (Table 3). However, the binomial logistic regression model presented in Table 4, shows that factors like gender and educational level have limited predictive value for urban sports participants. Only the age group of 46-65 years old appears to have a significant contribution to the predictive value of the model. This suggests that characteristics such as gender, age, and educational level do not primarily differentiate between urban and non-urban sports participants in our study.

Rather than demographics, urban sports participants appear to be distinguished from more 'traditional' sports participants by the location and the level of their sports participation and by their motives for sports participation. In this study, two definitions of urban sports are examined. One definition is based on specific urban sports activities, and another – a broader definition of urban sports– on the sports' location and the setting. Both in the descriptive statistics and in the binomial regression analysis, we find few differences between sports participants and the broader defined urban sports participants, suggesting that the trend towards more informal sports settings has blurred the lines between traditional and urban sports [2,12,13,14,15,16].

This trend towards informality is in line with larger shifts in sports participation, where even traditional sports are increasingly practised in non-organised settings. For instance, our results report recreational cycling, running, and walking as the three most practised sports in the city of Bruges [59]. All three of these sports were most of the time practised in the public space of the city and self- or non-organised settings and were therefore allocated as urban sports in the broader definition. Consequently, defining urban sports solely based on location and setting proves to be insufficient to distinguish between traditional and urban sports effectively.

In contrast, the definition based on specific urban sports seems more appropriate to create a profile of the urban sports participants and to examine the differences between 'more traditional' and urban sports participants. Based on this definition, the location of sports is the most distinguishing factor between urban sports and non-urban sports participants. Our findings support this distinction, as a significantly lower share (25.6%) of the total sports population practices their sports in a public location in comparison to the urban sports population (52.1%). This aligns with the established literature that describes public space as one of the two fundamental features of urban sports [33,34]. Surprisingly, the other fundamental feature of a self- or non-organisational setting has no significant value in predicting urban sports participation. Moreover, urban sports participants can be distinguished from more 'traditional' sports participants by their significantly higher participation in competitive and/or recreational levels in comparison to a solely recreational level. This more competitive nature of urban sports participants contradicts prior research. More specifically, according to Bruggeling & Van Baternburg-Eddes (2020) and Van der Meijde and colleagues (2022), urban sports are often characterised by a less competitive environment and urban sports participants value more the social aspect and the sense of community than the competitive aspect of sports [33,34].

To reconcile these – at first glance – conflicting findings, we draw on two concepts named *sportisation* and *sportification*. As aforementioned, *sportisation* involves integrating play-like activities into sports organisations, making them more competitive, standardised, and regulated [54,55]. A prime example of *sportisation* is the evolution of parkour from a self-organised and play-like activity to a structured sports discipline marked by weekly training sessions, formal associations, and dedicated sports facilities [52,60].

On the other hand, sportification describes the process of a non-organised sports activity evolving into a more institutionalised, formalised, and specialised form by adding components to an existing activity to increase its appeal [61,62,63,64,65,66]. The process of *sportification* can be divided into three basic mechanisms: the institutionalisation of practices, the formalisation of standards, and the specialisation of roles [67,68]. For example, 3x3 basketball originated as people playing basketball on public fields in the city in a non-organised way. In the past few years, 3x3 basketball underwent a lot of *sportification* resulting in world championships being organised and being integrated into the programme of the Olympic Games [31,50].

The two processes of *sportisation* and *sportification* are often used in different ways in the literature and their definitions are equivocally used. In fact, both processes are inextricably associated. The *sportisation* of an activity can be regarded as the process where a 'play-like' activity becomes a sport by being integrated into the organisation of sports [52,53,54,55]. The *sportification* process on the other hand can be seen as the non-organised sports activity evolving into a more institutionalised, formalised, and specialised form [62,63,64,65,67,68]. Often the *sportisation* process is followed by the *sportification* process when a play-like activity becomes a sports activity and evolves into the 'sportificated' form.

In our study, we observed that urban sports in Flanders, including Bruges, are subject to the processes of *sportisation* and sportification. External factors such as media and commercial

evolutions have fuelled *sportisation* of urban sports [69]. For example, the increasing use of urban sports in brand promotion is an important external factor for the *sportisation* process [22,25,26]. At the same time, the 'more traditional' sports organisations and institutions have embraced urban sports to enhance their offerings. Specifically, the 'more traditional' sports organisations and institutions use urban sports to improve the survival, safety, and accessibility of their 'sports branch' [69]. For example, in Flanders, sports federations have started to organise and offer training and competitions for urban sports activities and specific urban sports infrastructures are being built [70]. The Flemish Gym Federation (GymFed) incorporated parkour/freerunning in its programme with multiple competitions, events, and challenges [60]. In this way, parkour is incorporated into the sports organisations in Flanders and therefore underwent *sportisation*. Consequently, the competitive nature of urban sports has gained prominence.

Overall, in Flanders, the 'traditional' sports organisations and institutions – such as the Flemish sports administration and sports federations – are aware of the popularity of urban sports and the shift in sports participation. As a response, these organisations implement urban sports in their operations to increase the popularity of their 'sports branch' which is the *sportisation* process. However, the implementation of these urban sports is done according to the organisational principles and knowledge present in these 'more traditional' sports organisations [60]. Therefore, urban sports are mirrored by the more 'traditional' sports offer in terms of organisational setting and the competitive nature is gaining in importance which is *sportification* [69]. Because of these *sportisation* and *sportification* processes, urban sports in Flanders are not necessarily practised in a self- or non-organised setting and cannot be categorised as less competitive compared to the 'more traditional' sports. Nevertheless, this more competitive environment does not necessarily implicate that the social aspect and the sense of community in urban sports are lost. Anyway, the *sportisation* and *sportification* processes in urban sports are still evolving so further research on their evolution is needed.

Lastly, when examining the motives for sports participation, two of the four motives for sports participation have a significant contribution to explaining the difference between urban sports participants and non-urban sports participants. Urban sports participants indicate to prioritise less the physical motives of better health, better condition, and a better-looking body. In contrast, urban sports participants value more the fun aspect and the relaxation aspect of their sports activities than non-urban sports participants. This aligns with the defining characteristics of urban sports, which emphasise the fun, freedom, and physicality while rejecting the rigidity of traditional sports [19,20,21,22,23].

It is worth noting that some literature on urban sports reports the social aspect of sports to be a significant motivator for urban sports participants [30,33,34]. In our study, however, social motives have no significant value in predicting urban sports participation.

In conclusion, aiming to establish a comprehensive profile of urban sports participants, our results indicate rather conflicting findings with the literature. Existing literature often portrays urban sports participants as a rather homogeneous group of young, male individuals with lower educational backgrounds, practising their urban sports in a recreational way with social motives for practising [30,33,34,44,45]. Our findings, on the other hand, suggest urban sports participants to be a heterogeneous group of individuals since sociodemographic characteristics are not even considered a distinguishing factor between urban and traditional sports participants. These results might indicate that urban sports are attracting a wider range of sports participants than they used to and are therefore forming a more heterogenic group. This evolution might also be a result of the traditionalisation or institutionalisation of urban sports resulting from the *sportisation* and *sportification* processes as discussed earlier. This heterogeneity might be an important argument for governments and sports organisations to include urban sports in their policies as urban sports

present a valuable avenue to engage with a diverse range of current and prospective sports participants. On the other hand, whilst literature [30,44,48] indicated urban sports to be more inclusive and more attractive to vulnerable groups in society, the results in this paper suggest otherwise. Consequently, involving urban sports in policy programmes might not solve the lack of social inclusion in sports participation since the same, yet heterogenic, groups in society will be reached.

Conclusion

The dearth of knowledge concerning urban sports poses pressing challenges for local governments and sports organisations, especially considering its increasing popularity. In order to develop targeted policies to promote and facilitate urban sports, it is imperative to gain more insight into the characteristics, preferences, and main drivers of urban sports participants. Therefore, this research sought to compare urban and 'more traditional' sports participants in terms of demographic characteristics, sports participation behaviours, and the motives to participate in sports.

Our study indicates that sociodemographic characteristics of gender, age, and educational level do not explain the differences between urban and 'more traditional' sports participants. Instead, the differences can be explained by sports participation characteristics and motives for sports participation. Urban sports are characterised by two predominant factors: participation in public locations and a non-organised setting. In line with existing literature, public location appears to be the most distinguishing factor between urban and non-urban sports. On the other hand, the non-organised setting proves not to be significantly distinguishing. Contrary to prevailing literature, our findings suggest that urban sports participants exhibit more participation in a competitive context compared to non-urban sports participants.

These two contradictory findings might be partially explained by the *sportisation* process on the one hand and the *sportification* process on the other, which are exerting a profound influence on urban sports in Flanders. These processes are rapidly integrating urban sports disciplines in the Flemish sports environment, with numerous events being organised and urban sports branches being co-opted in the standard programmes of sports organisations.

In sum, these developments are transforming many urban sports into more traditional forms, thus accounting for the limited differences between urban and traditional sports participants.

In conclusion, this study aimed to establish a multifaceted profile of the urban sports participant and compare it with the traditional sports participant. While existing literature often depicted the group of urban sports participants to be rather homogenous, our results suggest a remarkable heterogeneity within this demographic. Ultimately, as urban sports continue to gain popularity, urban sports present an excellent avenue for governments and sports organisations to reach a wide range of sports participants and potential sports participants.

Limitations

The data of this paper was gathered in a large research project on sports participation in the city of Bruges. However, this survey had no specific questions on urban sports, thus limiting the depth of information available on urban sports participation. In addition, participants could not indicate whether they consider themselves urban sports participants or not.

Second, the limited presence of Bruges residents with a migration background and/or a low socio-economic status restricted our ability to explore the demographic differences between urban and ‘more traditional’ sports participants. Therefore, the specificity of the Bruges population renders it unsuitable for representing a broader range of cities.

Third, we utilised two distinct definitions of urban sports based on recent literature that provide a reasonably accurate approximation of urban sports. However, these definitions were not exhaustive nor conclusive since no unequivocal definition of urban sports is reported in the literature. This means we could have missed urban sports participants, and on the other hand, we could have misclassified non-urban sports participants as urban sports participants.

Further research

Further research should examine the differences between urban and 'more traditional' sports participants in migration background and socio-economic status.

Additionally, investigating the evolving nature of urban sports is a pertinent area of inquiry. Urban sports are the subject of *sportisation* and *sportification* driven by governments and sports federations. These processes continue to evolve, and the motives of urban sports participants in this transformation remain largely unknown. Therefore, further research should focus on uncovering the motives of urban sports participants within the context of sports evolution and explore the various settings of urban sports participation.

Acknowledgements

We would like to thank the members of the local sports authority in the city of Bruges for their help in gathering data and we would like to thank the respondents who took part in the online survey.

References

1. Eime R, Sawyer N, Harvey J, Casey M, Westerbeek H, Payne W. Integrating public health and sport management: Sport participation trends 2001–2010. Sport Management Review [Internet]. 2015 Apr 1;18(2):207–17. Available from: https://doi.org/10.1016/j.smr.2014.05.004
2. Scheerder, J., Claes, E., Vanreusel, B. Sportief (en) innovatief in een veranderende samenleving. Een verkennende annalyse van good practises als inspiratie voor de clubsport van de toekomst [Internet]. Beleid & Management in Sport Studies. KU Leuven/Departement Bewegingswetenschappen/Onderzoeksteam Sport in/for Society.; 2014 [cited 2023 Aug 4]. Available from: https://gbiomed.kuleuven.be/english/research/50000737/research/policy-in-sports-physical-activity-research-group/bms-studies/studies/bms022.pdf
3. The impact of COVID-19 on sport, physical activity and well-being and its effects on social development | DISD [Internet]. Available from: https://www.un.org/development/desa/dspd/2020/05/covid-19-sport/
4. Research for CULT Committee - EU sports policy: assessment and possible ways forward | Think Tank | European Parliament [Internet]. Available from: https://www.europarl.europa.eu/thinktank/en/document/IPOL_STU(2021)652251
5. Coalter F. A wider social role for sport [Internet]. Routledge eBooks. 2007. Available from: https://doi.org/10.4324/9780203014615
6. Eime R, Young J, Harvey J, Charity M, Payne W. A systematic review of the psychological and social benefits of participation in sport for children and adolescents: informing development of a conceptual model of health through sport.

International Journal of Behavioral Nutrition and Physical Activity [Internet]. 2013 Jan 1;10(1):98. Available from: https://doi.org/10.1186/1479-5868-10-98

7. Rakovac M, Pedisic Z. Physical activity and sport participation in the European Union. ResearchGate [Internet]. 2022 Jan 1; Available from: https://www.researchgate.net/publication/369065541_Physical_activity_and_sport_participation_in_the_European_Union
8. Scheerder, J., Thibaut, E. Studie over de Bewegingsactiviteiten in Vlaanderen (SBV) 1969-2019. Een halve eeuw sportparticipatie in cijfers [Internet]. Beleid & Management in Sport Studies. KU Leuven/Departement Bewegingswetenschappen/Onderzoeksteam Sport in/for Society.; 2021 [cited 2023 Aug 4]. Available from: https://gbiomed.kuleuven.be/english/research/50000737/research/policy-in-sports-physical-activity-research-group/bms-studies/studies/bms100-finaal.pdf
9. Scheerder J, Thibaut E. m.m.v. Willem, A. & Derom, I. Sportparticipatie in Vlaanderen [Internet]. 2022 [cited 2023 Aug 4]. Available from: https://media.sport.vlaanderen/m/3c0884e5fda45df0/original/Presentatie%20algemene%20resultaten%20Participatiesurvey%202020.pdf
10. Sports participation in the European Union – Mulier Instituut [Internet]. Mulier Instituut. ;2005. Available from: https://www.mulierinstituut.nl/publicaties/3045/sports-participation-in-the-european-union/
11. Scheerder J. Tofsport in Vlaanderen. Groei, omvang en segmentatie van de Vlaamse recreatiesportmarkt. F&G Partners [Internet]. 2007 Jan 1; Available from: https://lirias.kuleuven.be/handle/123456789/264241

12. Borgers J, Pilgaard M, Vanreusel B, Scheerder J. Can we consider changes in sports participation as institutional change? A conceptual framework. International Review for the Sociology of Sport [Internet]. 2016 Apr 4;53(1):84–100. Available from: https://doi.org/10.1177/1012690216639598
13. Deelen I, Ettema D, Kamphuis CBM. Sports participation in sport clubs, gyms or public spaces: How users of different sports settings differ in their motivations, goals, and sports frequency. PLOS ONE [Internet]. 2018 Oct 8;13(10):e0205198. Available from: https://doi.org/10.1371/journal.pone.0205198
14. Eime R, Charity M, Westerbeek H. The Sport Participation Pathway Model (SPPM): a conceptual model for participation and retention in community sport. International Journal of Sport Policy and Politics [Internet]. 2022 Feb 7;14(2):291–304. Available from: https://doi.org/10.1080/19406940.2022.2034913
15. Harris S, Nichols G, Taylor M. Bowling even more alone: trends towards individual participation in sport. European Sport Management Quarterly [Internet]. 2017 Mar 23;17(3):290–311. Available from: https://doi.org/10.1080/16184742.2017.1282971
16. Neal S, Pang B, Parry KD, Rishbeth C. Informal sport and leisure, urban space and social inequalities: Editors' Introduction. Leisure Studies [Internet]. 2023 Jan 11;1–12. Available from: https://doi.org/10.1080/02614367.2022.2162109
17. Tak I, Rutten J, Van Goeverden W, Barendrecht M. Sports participation and injury related to the COVID-19 pandemic: will data support observations from clinicians and athletes? BMJ Open Sport and Exercise Medicine [Internet]. 2022 Feb 1;8(1):e001317. Available from: https://doi.org/10.1136/bmjsem-2022-001317
18. Thibaut E, Constandt B, De Bosscher V, Willem A, Ricour M, Scheerder J. Sports participation during a lockdown. How COVID-19 changed the sports frequency and

motivation of participants in club, event, and online sports. Leisure Studies [Internet]. 2021 Dec 29;41(4):457–70. Available from: https://doi.org/10.1080/02614367.2021.2014941

19. Booth D, Thorpe H. Berkshire encyclopedia of extreme sports [Internet]. 2007. Available from: http://ci.nii.ac.jp/ncid/BA8226453X
20. Donnelly P. Sport as a site for "Popular" resistance. In: University of Toronto Press eBooks [Internet]. 1988. p. 69–82. Available from: https://doi.org/10.3138/9781442602861-007
21. Thorpe H, Ahmad N. Youth, action sports and political agency in the Middle East: Lessons from a grassroots parkour group in Gaza. International Review for the Sociology of Sport [Internet]. 2013 Jun 18;50(6):678–704. Available from: https://doi.org/10.1177/1012690213490521
22. Thorpe H, Wheaton B. 'Generation X Games', Action Sports and the Olympic Movement: Understanding the cultural Politics of incorporation. Sociology [Internet]. 2011 Oct 1;45(5):830–47. Available from: https://doi.org/10.1177/0038038511413427
23. Wheaton B. Introducing the consumption and representation of lifestyle sports. Sport in Society [Internet]. 2010 Sep 1;13(7–8):1057–81. Available from: https://doi.org/10.1080/17430431003779965
24. Wheaton B. Understanding lifestyle sport [Internet]. Routledge eBooks. 2004. Available from: https://doi.org/10.4324/9780203646069
25. Dilek C. Red Bull's accelerating sports marketing strategies [Internet]. Segmentify. 2023. Available from: https://segmentify.com/blog/red-bulls-accelerating-marketing-strategies/

26. Shakeri S. Red Bull influence in sports: What makes it unique? Bleacher Report [Internet]. 2017 Oct 3; Available from: https://bleacherreport.com/articles/34513-red-bull-influence-in-sports-what-makes-it-unique

27. Red Bull Athletes [Internet]. RedBull. [cited 2023 Aug 4]. Available from: https://www.redbull.com/be-nl/athletes?filter.countryCode=BE

28. Müller M. What makes an event a mega-event? Definitions and sizes. Leisure Studies [Internet]. 2015 Jan 13;34(6):627–42. Available from: https://doi.org/10.1080/02614367.2014.993333

29. Rinehart RE. Chapter 7 ESPN's X games: Contests of opposition, resistance, co-option, and negotiation. In: Research in the sociology of sport [Internet]. 2008. p. 175–95. Available from: https://doi.org/10.1016/s1476-2854(07)00207-5

30. De Bruijn M. Onderzoek urban sports Rotterdam: de kansen en uitdagingen voor gemeenten. Allesoversport.nl [Internet]. 2021 Jan 11; Available from: https://www.allesoversport.nl/thema/beleid/onderzoek-urban-sports-rotterdam-de-kansen-en-uitdagingen-voor-gemeenten/

31. Riding the Olympic wave : 3x3 basketball, BMX freestyle, breaking, skateboarding, sport climbing, surfing and much more : 17.03.2022 - 05.03.2023, The Olympic Museum, free entrance : press kit / The Olympic Museum [Internet]. Olympic World Library. Available from: https://library.olympics.com/Default/doc/SYRACUSE/1997834/riding-the-olympic-wave-3x3-basketball-bmx-freestyle-breaking-skateboarding-sport-climbing-surfing-a?_lg=en-GB

32. ZOOM IN - URBAN SPORTS [Internet]. Olympic World Library. Available from: https://library.olympics.com/default/urban-sports.aspx?_lg=en-GB

33. Onderzoek urban sports – Mulier Instituut [Internet]. Mulier Instituut. Available from: https://www.mulierinstituut.nl/publicaties/25707/onderzoek-urban-sports/
34. Kijk op urban sports – Mulier Instituut [Internet]. Mulier Instituut. Available from: https://www.mulierinstituut.nl/publicaties/27162/kijk-op-urban-sports/#:~:text=Urban%20sports%20is%20een%20verzamelnaam,doen%20vooral%20jongeren%20deze%20sporten.
35. Hoyng J. Urban sport is cool en hot. Allesoversport.nl [Internet]. 2020 Jan 10; Available from: https://www.allesoversport.nl/thema/beleid/urban-sport-is-cool-en-hot/
36. Het Brugs beleidsprogramma 2019-2024 [Internet]. Brugge. 2019. Available from: https://www.brugge.be/beleidsprogramma2019-2024
37. Beleidsplannen voor sport en beweging [Internet]. Stad Leuven. 2023. Available from: https://www.leuven.be/sportbeleid
38. Urban Sport - London Sport [Internet]. London Sport. 2022. Available from: https://londonsport.org/our-work/completed-projects/urban-sport/
39. Welkom | antwerpen.be [Internet]. A-stad. Available from: https://www.antwerpen.be/info/6220c1c0d7e19758f0442acf/sportbeleidsplan
40. Krachtlijnen Gents sportbeleid [Internet]. Stad Gent. 2020. Available from: https://stad.gent/sites/default/files/media/documents/41371-20200123_NO_Krachtlijnen%2520Gents%2520sportbeleid-79457b.pdf
41. Groot(s) op mensenmaat: bestuursakkoord 2019-2024 [Internet]. Hasselt heeft het. 2019. Available from: https://www.hasselt.be/sites/hasselt/files/documenten_stad_hasselt/bestuursakkoord_20190204_hasselt.pdf

42. Scheerder J. Huyghe J, Onderzoek naar sport- en beweegparticipatie in Brugge. Methodologie & representativiteit (deel 2) [Internet]. Beleid & Management in Sport Studies. KU Leuven/Departement Bewegingswetenschappen/Onderzoeksteam Sport in/for Society.; 2023 May [cited 2023 Aug 4]. Available from: https://gbiomed.kuleuven.be/english/research/50000737/research/pash/research-lines/sports-in-for-society/bms-studies/studies/bms122.pdf

43. Brussel en centrumsteden [Internet]. Departement Zorg. 2016. Available from: https://www.departementwvg.be/vipa-brussel-en-centrumsteden

44. User S. Moev zet je school in beweging [Internet]. MOEV. Available from: https://www.moev.be/urban-sports

45. Sport en bewegen in de openbare ruimte – Mulier Instituut [Internet]. Mulier Instituut. Available from: https://www.mulierinstituut.nl/publicaties/26297/sport-en-bewegen-in-de-openbare-ruimte/

46. Camoletto RF, Sterchele D, Genova C. Managing alternative sports: new organisational spaces for the diffusion of Italian parkour. Modern Italy [Internet]. 2015 Aug 1;20(3):307–19. Available from: https://doi.org/10.1080/13532944.2015.1065237

47. Sellering M. Urban sports: hoe sportende jongeren zichzelf organiseren en gemeenten dat kunnen stimuleren, ondersteunen en faciliteren. 2021.

48. Spaaij R, Broerse J, Oxford S, Luguetti C, McLachlan F, McDonald B, et al. Sport, Refugees, and Forced Migration: A Critical Review of the literature. Frontiers in Sports and Active Living [Internet]. 2019 Oct 11;1. Available from: https://doi.org/10.3389/fspor.2019.00047

49. Urban Sports site op stelplaats De Lijn in Assebroek: Stad stelt concessionaris aan [Internet]. Stad Brugge. Available from: https://www.brugge.be/urban-sports-site-op-stelplaats-de-lijn-in-assebroek-stad-stelt-concessionaris-aan
50. FIBA 3x3 World Cup 2022 [Internet]. FIBA.Basketball. Available from: https://www.fiba.basketball/nl/3x3WorldCup/2022
51. Sporza. WK breaking in Leuven: “Voor het plezier en voor olympische kwalificatiepunten.” sporza.be [Internet]. 2023 Apr 26; Available from: https://sporza.be/nl/2023/04/26/wk-breaking-in-leuven-voor-het-plezier-en-voor-olympische-kwalificatiepunten~1682517847849/
52. Larsen SH. The institutionalisation of Parkour in Denmark. A national case of how institutional isomorphism works and affect lifestyle sport. International Journal of Sport Policy and Politics [Internet]. 2022 Feb 27;14(3):401–17. Available from: https://doi.org/10.1080/19406940.2022.2043928
53. Wheaton B, O’Loughlin A. Informal sport, institutionalisation, and sport policy: challenging the sportization of parkour in England. International Journal of Sport Policy and Politics [Internet]. 2017 Jan 2;9(1):71–88. Available from: https://doi.org/10.1080/19406940.2017.1291533
54. Stebbins RA, Elias N, Dunning E. Quest for excitement: sport and leisure in the civilizing process. Canadian Journal of Sociology [Internet]. 1988 Jan 1; Available from: https://doi.org/10.2307/3340872
55. Maguire J. Global Sport: Identities, Societies, Civilizations. Polity; 1999.
56. Field AP. Discovering statistics using SPSS (and sex and drugs and rock “n” roll) (3rd edition). London: Sage [Internet]. 2009 Jan 1; Available from:

https://repository.tudelft.nl/view/MMP/uuid:3a9d01bb-83a9-4572-865a-87126ae60165/

57. Urban Sports (productverslag, Stichting Trudo Eindhoven). Rietveld, M.; 2008. Available from: https://brochures.nhtv.nl/scriptiebank/documenten/Urban%20sports-Rietveld,%20M..pdf
58. Agentschap Binnelands Bestuur | Gemeente-Stadsmonitor [Internet] 2022. Available from: https://gemeente-stadsmonitor.vlaanderen.be/
59. Huyghe J, Scheerder J. Onderzoek naar sport- en beweegparticipatie in Brugge. Resultaten van de stadssurvey (deel 3) [Internet]. Beleid & Management in Sport Studies. KU Leuven/Departement Bewegingswetenschappen/Onderzoeksteam Sport in/for Society.; 2023 May [cited 2023 Aug 4]. Available from: https://gbiomed.kuleuven.be/english/research/50000737/research/pash/research-lines/sports-in-for-society/bms-studies/studies/bms123.pdf
60. De Bock T, Scheerder J, Theeboom M, Schyvinck C, De Clerck T, Willem A. An organizational change perspective on the incorporation of parkour in a gymnastics federation. Sport in Society [Internet]. 2023 Jul 11;1–22. Available from: https://doi.org/10.1080/17430437.2023.2228732
61. Batuev M, Robinson L. What influences organisational evolution of modern sport: the case of skateboarding. Sport, Business and Management [Internet]. 2018 Nov 12;8(5):492–510. Available from: https://doi.org/10.1108/sbm-10-2017-0052
62. Cachay K. Versportlichung der Gesellschaft und Entsportung des Sport? System¬theoretische Anmerkungen zu einem gesellschaftlichen Phänomen. [Internet]. 2018. Available from: https://pub.uni-bielefeld.de/record/1940503

63. Crum B. The Sportification of the Society and the Internal Differentiation of Sport, in Proceedings of the First European Congress on Sport Management, 1st EASM Congress. Groningen: European Association of Sport Management. 1993 Sept.

64. Digel H. Die Versportlichung unserer Kultur und deren Folgen fuer den Sport - ein Beitrag zur Uneigentlichkeit des Sports [Internet] 1990. Available from: https://www.bisp-surf.de/Record/PU199103048109

65. Digel H. Sport in a changing society. Sociological Essays (Sport Science Studies 7) [Internet]. 1995 Jan 1; Available from: https://tubiblio.ulb.tu-darmstadt.de/1959/

66. Heere B. Embracing the sportification of society: Defining e-sports through a polymorphic view on sport. Sport Management Review [Internet]. 2018 Jan 1;21(1):21–4. Available from: https://doi.org/10.1016/j.smr.2017.07.002

67. Collinet C, Delalandre M, Schut PO, Lessard C. Physical practices and sportification: Between institutionalisation and standardisation. The example of three activities in France. International Journal of the History of Sport [Internet]. 2013 May 1;30(9):989–1007. Available from: https://doi.org/10.1080/09523367.2013.782538

68. Yang Z, Bai Y, Wei M. The importance of creativity in the sportification of breakdance. Frontiers in Education [Internet]. 2022 Mar 7;7. Available from: https://doi.org/10.3389/feduc.2022.855724

69. Batuev M. “Free sports”: organizational evolution from participatory activities to Olympic sports [Doctoral Thesis]. University of Stirling; 2015.

70. Thorpe H, Dumont G. The Professionalization of Action Sports: Mapping trends and future directions. Sport in Society [Internet]. 2018 Feb 21;22(10):1639–54. Available from: https://doi.org/10.1080/17430437.2018.1440715

Orignal Ethical approval lettre (dutch)

Click here to access/download
Supporting Information
Goedkeuring G-2022-4993-R7(AMD).pdf

Dataset

Click here to access/download
Supporting Information
Urban sport official dataset 8.06.sav

Official Ethical approval letter (english)

Click here to access/download
Supporting Information
Approval G-2022-4993-R7(AMD).pdf

Ethical approval email (dutch)

Click here to access/download

Supporting Information

Screenshot Ethical approval mail (dutch).png

Ethical approval email (english)

Click here to access/download
Supporting Information
Screenshot Ethical approval mail (english).png
