# Supplementary material for: Unveiling the urban sports landscape: Profiling participants, motives, and policy implications
Source: PLoS One. 2024 Jul 5;19(7):e0306305. doi: 10.1371/journal.pone.0306305 (PMC11226041; doi:10.1371/journal.pone.0306305)
Supplement: Attachment — Submitted filename: Rebuttal letter.docx [file pone.0306305.s003.docx]

**Dear editors,**

**We thank the reviewers for their valuable comments on the manuscript and have edited the manuscript to address their concerns and suggestions. Based on their suggestions, we believe the modifications we made enhanced the clarity and depth of the manuscript. A summary of the key modifications we made as response to the reviewers is provided below.**

- ***We condensed the introduction of the manuscript, included the urban sports part in this section and clarified some sections with additional references.***
- ***We explicitly discussed the validity of the methods we used to investigate the motives to participate in urban sports with additional information and references to national and international publications.***
- ***We restructured the conclusion section and added some practical implications derived from the findings of our study.***

**In the following section we will provide a detailed description of the adjustments we made in response to the comments or suggestions per reviewer.**

**Reviewer #1**: Dear authors, first of all, thank you for the submission made to Plos One. The authors seek to establish the profile of urban sports participants regarding their characteristics, behaviors and motivations for practicing sports in a specific region. Overall, the authors did a good job and the conclusions obtained are interesting. Still, there are some points that, if improved, could help the work reach its full potential.

Below you can find my comments in detail.

The introduction is well written and allows a clear understanding of the study problem. The methods are well described and allow replication of the procedures.
Regarding the results, considering and taking into account the typology of the study and the information that is intended to be emphasized, it would make perfect sense in my opinion that there are some graphic elements (figures, diagrams, etc.) that would complement the results.

*Thank you for this suggestion, we agree with your concern regarding the lack of graphic elements in the manuscript.*

*Although we seriously considered to add graphic elements or figures in the original manuscript, we specifically chose not to do so. The provided tables contain all necessary numbers and details to understand and interpret the results of our study. Given the current heaviness of the paper (cfr. comments by reviewer 2), we considered it already as rather exhaustive. In order to prevent the manuscript to become too expanded, we presumed it not preferable to add figures merely for repetition of the already provided data.*

In addition, taking into account the results, perhaps a point with practical recommendations derived from the findings would make sense. This is because the results can be useful and provide relevant information that can encourage the practice of physical activity.

Thank you for this suggestion. We agree that practical implications are an added value for this manuscript. Therefore, we added the following five practical implications on p. 34 and p. 35:

“*Firstly, our study challenges the presumption that urban sports are inherently more inclusive. The demographic characteristics of urban sports participants do not significantly differ from those of traditional sports participants. Consequently, integrating urban sports into policy programmes may not address the issue of social inclusion in sports participation, as it appears that the same, yet heterogeneous, groups in society are being reached.*

*Secondly, the increasing popularity and the heterogeneity of the urban sports participants present an opportunity for (local) governments and sports organisations. Urban sports can be an effective tool to reach a broad spectrum of people, engaging both current and prospective sports participants across various backgrounds and interests.*

*Thirdly, the rise in popularity of urban sports is closely linked to the trend towards informal sports participation. Governments should consider enhancing and facilitating informal sports settings to encourage those who do not engage with traditional sports organisations or clubs.*

*Fourthly, many urban sports have evolved into more traditional and formalised activities. It is important to recognise that urban sports participants may not necessarily resist traditional sports and their formalised structures. Acknowledging the shift towards organised and competitive formats within urban sports is crucial for accurately understanding their development.*

*Lastly, the assumption that urban sports environments are less competitive than traditional sports is increasingly inaccurate. Urban sports are frequently practised in competitive contexts, with organised competitions occurring globally.”*

**Reviewer #2**: The present study aimed to analyse whether sociodemographic characteristics of gender, age, and educational level do explain the differences between urban and traditional sports participants in Bruges, Belgium. Despite being interesting, the paper is very heavy, and becomes exhaustive to read. I consider that the manuscript could be more objective with a reduction of some sections. However, I hope that some of my concerns, that were identified further, after being solved, could contribute to fix it.

Introduction
Despite well designed, this section is longer. Urban sports section should be eliminated and add only a sentence/paragraph about urban sports in this section.

*We understand your concern regarding the length and density of the paper. We have carefully considered your suggestion to streamline the content for better readability and objectivity.

Therefore, we have revised the 'introduction' and 'urban sport' sections. We have condensed certain sections to ensure that the paper is more succinct and focused, while retaining the essential information (see track changes in the introduction). Additionally, we have removed the Urban Sports section as per your recommendation and integrated relevant information about urban sports into the introduction (p. 5-7).*

*We believe that these revisions have improved the clarity and flow of the manuscript, making it more accessible to the readers while maintaining the integrity of the research findings.*

The authors wrote “Although sports participation in Western Europe has increased constantly over the past decades, this trend appears to have stagnated in recent years [7,8,9,10].” However, bibliographic reference 10 is of 2005.

*We understand that this reference is outdated and has no additional value to this section nor other sections in the manuscript. Therefore, and because the other references suffice, we decided to remove this bibliographic reference.*

Methods
Data collection section identified that data were collected in Bruges, however, previous there was indication of the Flanders region. It is important to uniformise all document.

*Thank you for this valuable comment. We understand your concern and modified several sections to uniformise the manuscript.*

*Since the trends and evolutions of urban sports we discuss take place in all Western European countries, in the introduction we focus on the broader picture of all Western Europe. However, the data we collected in this study is specifically focused on Bruges (Flanders). Because Bruges can be best compared with other cities in Flanders, in the methodology and result sections we focus on the Flanders region separately. On page 8 we provided a new section in which we discuss the research context. This is presumed necessary to explain the rather complex political setting in Belgium when it comes to sports policy.*

My major concern with this study is related to the methods adopted by the authors to assess the motives. Why didn’t the author use a validated scale? It’s a huge fragility of this work to utilise this methodology. Beyond it, why not provide us with a methodology with more robustness to validate this option?

*Thank you for this insight on the lack of explanation on the validity of the scale used for assessing the motives. We acknowledge the fact that we didn’t substantially prove the validity of this scale in the original manuscript. Nevertheless, the scale we used to assess the motives is a validated scale used in several research projects and international publications. In the methodology section of the manuscript (p. 10), we included a section that discusses the validity of this scale based on (inter)national literature (n=6) ranging from 2005 until 2023. The motives stem from a scale developed by De Bourdeaudhuij et al. (2005) that has later been actualised and further validated in Dutch (Scheerder, Borgers & Willem, 2015; Thibaut, Scheerder, Derom & Willem, 2023) and international (Janssen et al., 2020; Thibaut et al., 2016) peer-reviewed books and journals. We hope this clarifies the validation of the methods we used.*

Results
Table 4 needs to be more descriptive. What means s.e.; ref, per example.

*Thank you for this comment. We tried to optimise the readability of table 4 by adding additional information of the terms we used below the table. (p. 24)*

Conclusion
This section has info that should be include in previous.

*Thank you for this insight. We restructured and optimised this section and made the conclusion section more succinct.*

We need of the practical implications of this study.

Thank you for this suggestion. We agree that practical implications are an added value for this manuscript. Therefore, we added the following five practical implications on p. 34 and p. 35: (cfr. Reviewer 1)

“*Firstly, our study challenges the presumption that urban sports are inherently more inclusive. The demographic characteristics of urban sports participants do not significantly differ from those of traditional sports participants. Consequently, integrating urban sports into policy programmes may not address the issue of social inclusion in sports participation, as it appears that the same, yet heterogeneous, groups in society are being reached.*

*Secondly, the increasing popularity and the heterogeneity of the urban sports participants present an opportunity for (local) governments and sports organisations. Urban sports can be an effective tool to reach a broad spectrum of people, engaging both current and prospective sports participants across various backgrounds and interests.*

*Thirdly, the rise in popularity of urban sports is closely linked to the trend towards informal sports participation. Governments should consider enhancing and facilitating informal sports settings to encourage those who do not engage with traditional sports organisations or clubs.*

*Fourthly, many urban sports have evolved into more traditional and formalised activities. It is important to recognise that urban sports participants may not necessarily resist traditional sports and their formalised structures. Acknowledging the shift towards organised and competitive formats within urban sports is crucial for accurately understanding their development.*

*Lastly, the assumption that urban sports environments are less competitive than traditional sports is increasingly inaccurate. Urban sports are frequently practised in competitive contexts, with organised competitions occurring globally.”*

**Reviewer #3:** The authors of this article did a great job demonstrating the relevance of their work to society. Furthermore, the article meets the magazine's standards, in line with what the magazine preaches in its values. However, I believe that there are some things that could give more strength and importance to the article and that can be considered or changed by the authors when resubmitting the article for publication. This way, I left my comments in a PDF file of the article, making it easier to specifically locate my considerations in the article.

*P. 5:
'It would be interesting to bring population data on how many people perform urban sports in relation to the general population and the importance of your study in relation to this and what is the gap in your work, since the gap in the literature demonstrates the strength, identity and importance of the study.'*

*Thank you for your insightful comment regarding the need for population data on urban sports participation and its relevance to our study. However, there is a current dearth of specific figures or rates globally, nationally, and locally concerning urban sport participation. Making it impossible to include population data on urban sport participation. Moreover, this lack of numbers is further complicated by the absence of a universally accepted definition for urban sports, making it challenging to quantify participation accurately.

However, we aimed to underscore the importance of urban sports through the contemporary significance that policymakers attribute to urban sports, particularly in larger urban centers (p. 5). More specifically, numerous cities worldwide are proactively adapting their sports policy strategies to accommodate the growing popularity of urban sports. For instance, Paris and Barcelona have taken notable steps to integrate parkour and skateboarding into their urban planning initiatives, indicative of the recognition of urban sports' cultural and recreational value.

Our study aims to address the gap in academic research on urban sports by shedding light on the profile, preferences, and motivations of participants. By doing so, we seek to provide insights crucial for the development of targeted policies aimed at promoting and facilitating urban sports. By understanding the dynamics driving urban sports participation, policymakers can better allocate resources and design initiatives that cater to the needs and interests of urban sports enthusiasts.*

*In sum, while the current lack of empirical data presents a challenge, it also underscores the importance and timeliness of our research by highlighting the complexities of urban sport participation, we aim to contribute valuable knowledge that can inform evidence-based policymaking and enhance inclusivity and accessibility of urban sport opportunities.*

P. 6:

**'***It would be interesting to bring in numbers (data) how much these sports bring benefits to the government in terms of economic gain from their commercialization and how many people are encouraged to perform physical activity through these sports.'*

*We have carefully considered your suggestion to include quantitative data on the economic benefits and the encouragement of physical activity through urban sports.

While it is currently impossible to isolate the SROI specifically attributable to urban sports due to current limitations in the literature, we have integrated (p. 3) relevant data that underscores the significant societal benefits of sport more broadly. The SROI framework helps to quantify these benefits, encompassing various aspects such as health improvements, enhanced social inclusion, and increased subjective well-being.

Although specific data for urban sports alone is not currently available, the high level of commercialization and their integration into policy plans in Western Europe (discussed throughout the introduction section) suggest a significant contribution to the overall SROI. These insights align with the assumption that urban sports play a vital role in encouraging physical activity and generating economic benefits.*

P.14: *'Most of the sports mentioned are practiced by younger people. Isn't there a limitation of the article in the statement that older people practice fewer of these sports? If so, it would be interesting to include it as a limitation of the article.'*

*Thank you for this comment. The main focus of our study is to examine differences between practitioners of urban sports and practitioners of traditional sports. In traditional sports older people are also proven to be less practicing. We therefore do not consider this a limitation, however, we acknowledge the distorted image this may give about older people practicing urban sports. For this reason, we tried to emphasise that we just try to compare urban with traditional sports participation in the discussion and conclusion, therefore not examining for relative underrepresentation of this older demographic in both of these sports practices.*

*Ultimately, our results show an underrepresentation of 46-65 years old people among the urban sports practitioners, but no significant differences between urban and traditional sports participants among the group of 66-75 years old people.*

*We explicitly discuss this on p.17:*

*“Compared to the overall sports population, urban sports participants – defined by specific sports –are significantly more frequently men (60.8%) and tend to be younger (12.9% aged 6-12 years, 14.0% aged 13-18 years, 24.6% aged 19-30 years). The age group of 46-65 years is significantly underrepresented among urban sports participants.”*

P.17: **'***If this data exists, it would be interesting to stratify the data in a table according to the types of street sports'*

*Thank you for this interesting comment. It would be very insightful to be able to distinguish between the different types of urban or street sports. Unfortunately, we are not able to do so because we don’t have the right data. Nevertheless, it is a very interesting research question for further research, so we added this suggestion to suggestions for further research as follows:*

*“Thirdly, we utilised two distinct definitions of urban sports based on recent literature to approximate urban sports participation. However, these definitions are neither exhaustive nor conclusive due to the lack of an unequivocal definition in the literature. This could have led to both the misclassification of non-urban sports participants as urban sports participants and the omission of true urban sports participants. Therefore, further research should aim to uncover the traits of various urban sports types rather than generalising the characteristics of urban sports as a whole. This nuanced understanding can help tailor interventions and support for diverse urban sport activities.“ (p.36).*

*P.24:* **'***Street sports in a broader perspective, as you say, could be considered as sporting events, or better yet, physical activity. Because, unlike physical exercise or sport itself, physical activity is a broader form of body movement. Physical exercise demands systematization and sport demands rules established by an organization, so the confusion of these terms can be a confusing factor in your study. Street sports, which originate in public spaces, are not necessarily carried out in public spaces, they are sports (with institutionalized rules) that can be carried out in private spaces, by companies that offer training in these sports. Therefore, it would be better for you to present it in a more specific way, which could be by modalities, through a theoretical framework that identifies sports as you did (better if it were all street sports) and differentiates from the broader perspective of street sports. street as physical activity.*

*Thank you for this insight. The term ‘street’ sports is quite uncommon in literature where most of the time ‘urban’ sports, ‘action’ sports or ‘lifestyle’ sports are used. However indeed there is a difference between those categories with urban sports being very similar to street sports. We added a small section in the introduction (p.5) where we introduce the term ‘street’ sports based on the following reference:*

*Engell, Z., Larsen, S. H., & Elmose-Østerlund, K. (2023). Participation in street sports-a national study of participation patterns among youth and adults. European Journal for Sport and Society, 1-24.ISO 690.*

*However, to avoid confusion between multiple terms we continued with urban sports as it is the main topic of our manuscript.*

The issue of physical activity versus physical exercise is indeed rather confusing. U*rban sports are crossing the thin line from physical activity to physical exercise because of the sportification and sportisation processes influencing its evolution. In the introduction and in the discussion of the manuscript we tried to clarify the sections about this evolution trough these processes (p. 7, 30-32).*

*The original forms of urban/street sports are indeed categorised as physical activity. These institutionalised forms of urban/streets sports undergoing the sportification and/or sportisation processes are more correctly categorised as physical exercise. However, this is not the main topic of our study since we mainly focus on drawing a profile of the urban sports participants. Therefore, and to avoid confusion, we decided not to go more in detail on the differences between physical activity and physical exercise within urban sports participation.*

*P27: 'You bring important population data and don't argue at the end. In general, the population has more active women than men, why did your study show that men practice more street sports than women? Why do people with high education practice these sports more, given that there is a study that shows the opposite result? Finally, why do older people practice these sports less? Bringing these factors in will show more strength in your results and in the study.'*

*Thank you for this valuable insight. We added a section in the discussion of our results on p. 27. In this section, as you suggested, we discuss the results of our demographic data and argue these with recent findings in literature.*

*Although our study shows no significant differences between urban and traditional sports participants in terms of educational level, men do appear to practice more urban sports compared to women. Also, in terms of age there are differences between urban sports participants and traditional sports participants in the constatation that urban sports participants are generally younger.*

*Recent findings in literature claim to have found an explanation for the differences in gender and age based on typical urban sports environment characteristics. On p. 27 of the manuscript we describe these findings, who can also explain for the differences in competitiveness, which are in contrast with findings in former literature.*

*P29:* **'***I believe that as a limitation, there would be the factor of not controlling what hinders the practice of physical activity in people who do little sport (such as violence, weather, lack of nearby places to practice, lighting, beauty of the place where they practice...). ‘*

*Thank you for this insight. After consideration we believe that this could indeed be a factor. Although we believe this could only mildly influence the study, it definitely deserves a place in the limitation section of the manuscript. Therefore, we added it as a fourth limitation of the study as follows:*

*“Lastly, this study did not gather information on potential barriers to sports participation, which may differ significantly between urban and traditional sports participants. Factors such as violence, weather, and lack of nearby infrastructure were not controlled for, which could influence the findings related to sports participation. Understanding these barriers is crucial to accurately assess and address the needs of both urban and traditional sports participants.”*

We thank the reviewers for their valuable comments. The revision we made based on the comments we discussed above, were indeed necessary to optimise the manuscript. The improved version of the manuscript is more succinct and is better substantiated for unveiling the urban sports landscape as is the title of the paper.

Kind regards,

Jef Huyghe

Nathan D’Hoore

Erik Thibaut

Jeroen Scheerder

Departement of Movement Sciences, KU Leuven, Leuven, Belgium
